# Transcriptomic analysis of equine chorioallantois reveals immune networks and molecular mechanisms involved in nocardioform placentitis

**DOI:** 10.1186/s13567-021-00972-4

**Published:** 2021-07-08

**Authors:** Hossam El-Sheikh Ali, Shavahn C. Loux, Laura Kennedy, Kirsten E. Scoggin, Pouya Dini, Carleigh E. Fedorka, Theodore S. Kalbfleisch, Alejandro Esteller-Vico, David W. Horohov, Erdal Erol, Craig N. Carter, Jackie L. Smith, Barry A. Ball

**Affiliations:** 1 Maxwell H. Gluck Equine Research Center, Department of Veterinary Science, University of Kentucky, Lexington, KY 40546 USA; 2 Theriogenology Department, Faculty of Veterinary Medicine, Mansoura University, Mansoura, 35516 Egypt; 3 UK Veterinary Diagnostic Laboratory, University of Kentucky, Lexington, KY 40546 USA; 4 Department of Biomedical and Diagnostic Sciences, University of Tennessee, Knoxville, USA

**Keywords:** Equine, Nocardioform placentitis, Chorioallantois, Transcriptome, *Amycolatopsis* spp.

## Abstract

Nocardioform placentitis (NP) continues to result in episodic outbreaks of abortion and preterm birth in mares and remains a poorly understood disease. The objective of this study was to characterize the transcriptome of the chorioallantois (CA) of mares with NP. The CA were collected from mares with confirmed NP based upon histopathology, microbiological culture and PCR for *Amycolatopsis *spp. Samples were collected from the margin of the NP lesion (NPL, *n* = 4) and grossly normal region (NPN, *n* = 4). Additionally, CA samples were collected from normal postpartum mares (Control; CRL, *n* = 4). Transcriptome analysis identified 2892 differentially expressed genes (DEGs) in NPL vs. CRL and 2450 DEGs in NPL vs. NPN. Functional genomics analysis elucidated that inflammatory signaling, toll-like receptor signaling, inflammasome activation, chemotaxis, and apoptosis pathways are involved in NP. The increased leukocytic infiltration in NPL was associated with the upregulation of matrix metalloproteinase (*MMP1*, *MMP3*, and *MMP8*) and apoptosis-related genes, such as caspases (*CASP3* and *CASP7*), which could explain placental separation associated with NP. Also, NP was associated with downregulation of several placenta-regulatory genes (*ABCG2*, *GCM1*, *EPAS1*, and *NR3C1*), angiogenesis-related genes (*VEGFA*, *FLT1*, *KDR*, and *ANGPT2*), and glucose transporter coding genes (*GLUT1*, *GLUT10*, and *GLUT12*), as well as upregulation of hypoxia-related genes (*HIF1A* and *EGLN3*), which could elucidate placental insufficiency accompanying NP. In conclusion, our findings revealed for the first time, the key regulators and mechanisms underlying placental inflammation, separation, and insufficiency during NP, which might lead to the development of efficacious therapies or diagnostic aids by targeting the key molecular pathways.

**Supplementary Information:**

The online version contains supplementary material available at 10.1186/s13567-021-00972-4.

## Introduction

Nocardioform placentitis (NP) is defined as a focal mucoid placental inflammation in which the bacterial infection is limited to the chorionic surface of the ventral placenta without infection of the fetus [1, 2]. NP was first diagnosed in central Kentucky (KY) in 1986 [3] with subsequent epizootics occurring there in 1998, 1999, 2011, 2017, and 2020 [1, 4]. Cases of NP have also been reported sporadically in Florida [5], South Africa [6], Italy [7] and most recently in New Zealand [8]. NP is characterized by late-term abortions, premature foals, neonatal deaths, and weak foals at term associated with an apparent fetal growth retardation due to placental separation and insufficiency with large areas of the chorion that may be involved with the lesion [1]. NP lesions may also be seen in the CA in mares with normal neonates. The distribution of the placental lesion in NP is distinct from those of ascending bacterial placentitis with lesions of NP mainly distributed in the cranial-ventral portion of the placenta near the junction of the uterine horns and body [1]. The lesion is often sharply demarcated from the surrounding normal placenta, and the affected placenta is covered with a thick, tan mucoid material [1, 2]. Histologically, the chorioallantois may demonstrate infiltration of neutrophils, lymphocytes, and macrophages with hyperplasia of the chorionic epithelium [1]. The surface exudate contains sloughed epithelial cells, leukocytes and an eosinophilic, amorphous material. Centrally, the chorionic villi are blunted and atrophied with lymphocytic infiltrates [1].

Nocardioform placentitis is associated with gram-positive, branching actinomycetes including *Amycolatopsis* spp., and *Crossiella equi* along with more recently characterized isolates of *Streptomyces atriruber* and *Streptomyces silaceus*, among others [9]. Characterization of actinomycetes associated with abortions during the 2011 outbreak of NP in central Kentucky revealed that *Amycolatopsis spp*. (49% of cases) was the most common, with *Crossiella equi* (29% of cases) as the next most frequent isolate [10].

To date, the pathogenesis of the disease remains poorly understood. Attempts to induce the infection in mares by intrauterine inoculation of *Crossiella equi* at the time of breeding or in pregnant mares via oral, intravenous, and intranasal routes with *Crossiella equi* were unsuccessful [11]. Like pathogenesis, the ecology and biology of the causative organisms, *Crossiella equi* and *Amycolatopsis *spp*.* remain unknown, as these organisms have only been isolated from affected placentae in mares. Additionally, the pathophysiology of the NP and the key regulators underlying placental inflammation, separation and insufficiency during NP remains unclear. These gaps in our knowledge about the disease are limiting the advancement toward efficient diagnostic, prevention and treatment protocols for NP.

The application of high-dimensional biology, such as RNA-sequencing could aid in revealing the NP transcriptomic signature, identifying the immune networks and pathways involved in NP with consequent elucidation of the pathophysiology of the disease. The better understanding of these key regulators and mechanisms holds potential for the development of new diagnostic tools and therapies to forestall NP-induced preterm labor. Therefore, the current study aimed to compare the transcriptomic changes in the CA of mares with NP with tissues collected from unaffected regions of chorioallantois as well as gestationally age-matched control CA.

## Materials and methods

### Experimental design

**Figure 1:**
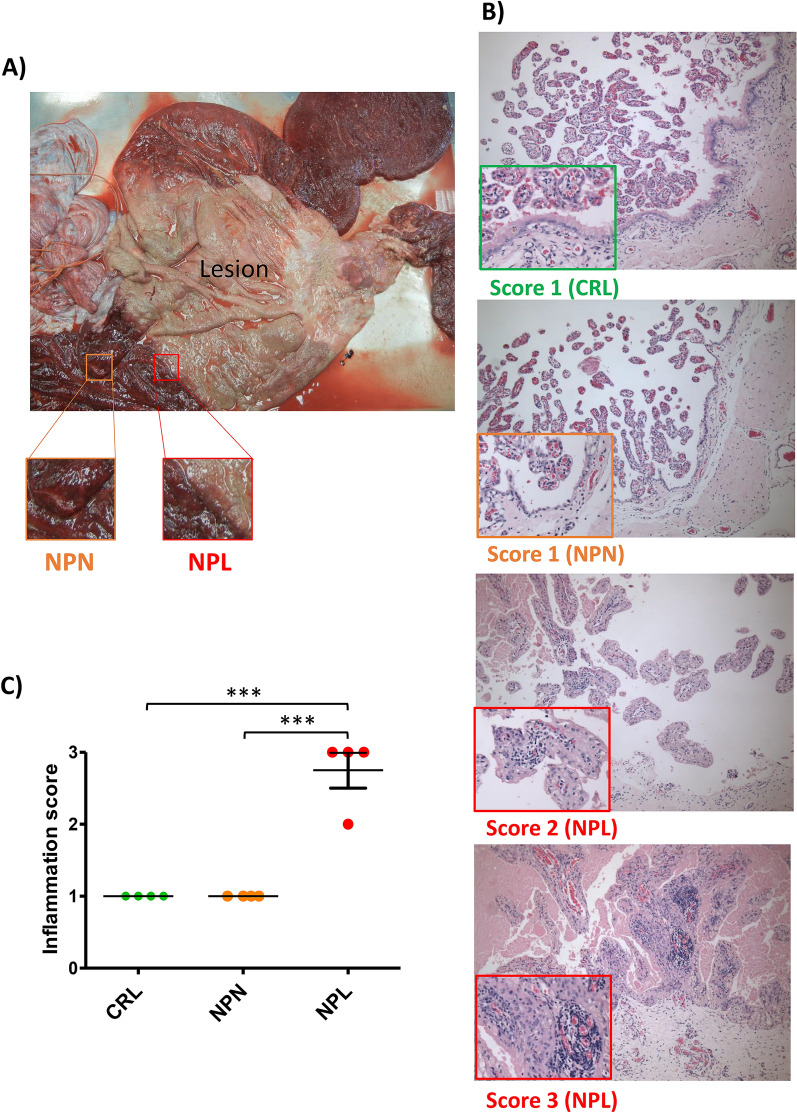
**Sampling sites from the nocardioform placentitis group and the histopathological evaluation of inflammation score.** **A** In the nocardioform placentitis group we collected chorioallantois samples from two sites. The fist sampling site (NPL) was at the margin of the nocardioform placentitis lesion. The second sampling site (NPN) was chosen where the chorionic surface is red and velvet in appearance with no gross evidence of pathology. **B**, **C** The inflammation score in the different sets (CRL, NPN, and NPL) and reprehensive H&E images of the different inflammation scores. The inflammation was graded as score zero (no inflammation; no inflammatory cells), score 1 (mild inflammation; few leukocyte infiltration), score 2 (moderate inflammation; moderate leukocyte infiltration), and score 3 (marked inflammation; large numbers of inflammatory cells).

During the 2017 foaling season, thoroughbred mares in central KY with suspected nocardioform placentitis had placental tissues collected at the time of foaling or abortion for subsequent histopathologic evaluation as well as RNA isolation. Within three hours of foaling/abortion, two samples were taken from the CA at the margin of the NP lesion (NPL, Figure 1A) using an 8-mm biopsy punch. One sample was fixed in 10% formalin, and one sample was preserved in RN = Alater® (#AM7021; Invitrogen). In addition, two samples were also taken from a normal-appearing region of the CA (NP normal; NPN, Figure 1A) over the uterine body and preserved as mentioned above. Tissues were handled carefully to avoid contamination with soil, manure, bedding or other foreign material. The remaining CA was submitted to the University of Kentucky Veterinary Diagnostic Laboratory for complete gross and histopathologic evaluation, as well as microbiology and PCR for *Amycolatopsis spp.* and *Crossiella equi* [10]. Among the collected cases, four cases (NPL = 4 and NPN = 4) were confirmed as Nocardioform placentitis (*Amycolatopsis spp.*) based upon microscopic examination of the placenta (presence of gram-positive branching bacilli) and confirmation by PCR [10]. Additionally, normal postpartum placenta (Control; CRL, *n* = 4) were collected from four normal foaling mares, and these placentae were also examined at the UKVDL to confirm normal placenta based upon pathological and microbiological examination.

### Total RNA extraction

Total RNA was isolated from all CA samples using the RNeasy Mini Kit (#74,104; Qiagen), and DNA digestion was performed on-column using RNase-free DNase I (#79,254: Qiagen), followed by cleanup procedures. All procedures were performed according to the manufacturer’s instructions. After extraction, RNA concentration and quality were analyzed using a Nanodrop 2000 spectrophotometer (#ND-2000; Thermo Fisher Scientific) and the Bioanalyzer® (Agilent, Santa Clara, CA, USA). All samples had a 260/280 ratio > 2.0 and RNA integrity number (RIN) > 9.0.

### Next-generation RNA sequencing (RNA-seq)

Next-generation RNA-seq was performed at the University of Louisville Center for Genetics and Molecular Medicine as described elsewhere [12]. In brief, the mRNA libraries were prepared from total RNA using TruSeq® Stranded mRNA Library Prep (#20,020,594; Illumina). Libraries were diluted to 10 nM, pooled, further diluted and denatured to single strand and run on a NextSeq 500 v2 (Illumina, San Diego, CA, USA) 300 cycle, High Output kit in a 2 × 150 bp PE read.

### Bioinformatics pipeline

Reads were trimmed for quality and adapters with TrimGalore 0.4.3, then mapped to EquCab3.0 using STAR 2.5.3a [13]. Expression values (Fragments Per Kilobase of transcript per Million fragments mapped; FPKM) of mapped reads were quantified using Cufflinks 2.2.1 [14] with the ENSEMBL annotation (ENSEMBL v.88). Differentially expressed genes (DEGs) were evaluated using Cuffdiff 2.2.1 based upon a false discovery rate (FDR) adjusted *p*-value < 0.05 after Benjamini–Hochberg correction for multiple-testing [14]. The DEGs were represented as heatmaps using the R-package “gplots” [15]. The overlap between DEGs in the NPL vs. CRL, NPL vs. NPN and NPN vs. CRL was illustrated with Venn diagrams using BioVenn and UpSet. The current RNA-seq data was deposited in the Gene Expression Omnibus (GEO; GSE154637) repository. The bioinformatics and functional genomics pipeline is summarized and illustrated in Additional file 1.

### Functional genomics

To investigate the biological functions of DEGs, DAVID Bioinformatics Resources version 6.8 along with the PANTHER pathways version 13.0 were used to functionally annotate genes based on gene ontology (biological process and pathways).

To predict upstream regulators relevant for each set of DEGs, upstream regulator analysis was carried out using Ingenuity pathway analysis (IPA, 2018) as described elsewhere [12]. To investigate the interaction and relationships between the potential upstream regulators, all known protein–protein interactions were referenced and matched using String version 10.5. The resultant interaction networks were visualized using Cytoscape version 2.8.3.

Weighted gene co-expression network analysis was carried out using WGCNA version 1.66 package in R [16] to construct gene co-expression networks as described elsewhere [17]. Gene co-expression clusters were generated from the all DEGs in NPL as described elsewhere [12]. In brief, following the construction of a matrix of pairwise correlations between all pairs of genes across the samples (*n* = 12), a weighted adjacency matrix was generated by raising co-expression similarity to a power β = 9 as determined for these sample sets. Then, a topological overlap matrix (TOM) was assembled and used as input for hierarchical clustering analysis. Then, a dynamic tree-cutting algorithm was used to identify gene modules or clusters (i.e., genes with high topological overlap). Gene clusters were visualized by the heatmap plot (TOM plot) of the gene network topological overlap. Module relationships were summarized by a hierarchical clustering dendrogram and TOM plot of module eigengenes (MEs). The associations between the gene clusters and the score of the trait of interest (i.e., inflammation score for each sample) were tested by correlating MEs to trait score. GO analysis (biological process) was performed on gene lists derived from module of interest. Module memberships (MM, i.e., the correlation between each gene’s expression profile with the ME of a given module as an indicator of the intramodular connectivity) and gene significance (GS, i.e., the correlation between the gene expression profile (FPKM) and the trait score (inflammation score) as a measure of biological relevance) were calculated [18]. The genes (network nodes) having MM ≥ 0.90, *p*-value < 0.05 and GS ≥ 0.6 were identified as intramodular hub genes [19].

To further investigate the cross-talk between DEGs in NP, we matched our DEGs with the available ligand–receptor pairs in the FANTOM5 database for protein-coding genes [20]. This dataset includes 2557 ligand-receptor interactions.

### Validation of RNA-seq using quantitative RT-PCR

For gene expression analysis, total RNA was reverse transcribed using TaqMan™ Reverse Transcription Reagents (#4,368,814; Invitrogen™). RT-qPCR was performed using PowerUp™ SYBR™ Green Master Mix (#A25741; Applied Biosystems™) along with specific primers designed for a subset of six selected targets using Primer-BLAST (NCBI) as described in Table 1, with actin beta (*ACTB*) and glucuronidase beta (*GUSB*) as reference genes based on Normfinder software (version 0.953) as described elsewhere [21]. PCR efficiency was evaluated using LinRegPCR (version 11.0) to ensure that all evaluated transcripts resulted in a PCR efficiency between 1.8 and 2.2 [22]. Delta CT (ΔCT) values calculated where ΔCT = (CT values of mRNA of interest—CT value of the reference genes). Results are presented as -ΔCT. One-way ANOVA was used to evaluate the significance of any changes in mRNA expression of tested targets between CRL, NPN and NPL groups, followed by a pairwise comparison of means using unpaired T-test. Differences of *P* < 0.05 were considered as statistically significant and any difference of 0.1 > *P* ≥ 0.05 was considered a trend. Pearson’s correlation was used to examine the correlation between mRNA expression results from RT-qPCR (−ΔCT) and the RNA-sequencing (normalized FPKM: Log_2_[FPKM + 1]) for each transcript. Descriptive statistics are expressed below as the mean ± standard error of the mean (SEM).

**Table 1:** **Forward and reverse primer sequences used for RT-qPCR analysis**

Gene	Forward	Reverse	Accession	Amplicon size (bp)
*IL1β*	CAGTCTTCAGTGCTCAGGTTTCTG	CATTGCCGCTGCAGTAAGT	XM_001495926.4	84
*IL1RN*	GCCTGTGTCAAGTCTGGTGA	ACTCTTTGGGCTTGTTGGTG	XM_005599766.3	217
*IL6*	GGATGCTTCCAATCTGGGTTCAAT	TCCGAAAGACCAGTGATGATTTT	NM_001082496.2	65
*PLAC8*	GTTTCATGTGCCTTGCGTTGT	ATATCGGGTCCGGTAGAGGG	XM_003364673.4	98
*SAA1*	CCTGGGCTGCTAAAGTCATC	AGGCCATGAGGTCTGAAGTG	XM_023646155.1	169
*INHBA*	CCTCCTCTTCCTTCTTCTTCTTCTT	GCAAGAGCTCCCTGGATATT	XM_023638729.1	99
*GUSB*	GGGATTCGCACTGTGGCTGTCA	CCAGTCAAAGCCCTTCCCTCGGA	XM_001493514	117
*ACTB*	CGACATCCGTAAGGACCTGT	CAGGGCTGTGATCTCCTTCT	NM_001081838	100

Primers were generated using Primer-BLAST (NCBI).

Key: RT-qPCR, Real time quantitative polymerase chain reaction; NCBI, National Center for Biotechnology Information; *IL1β*, Interleukin 1β; *IL1RN*, Interleukin 1 Receptor Antagonist; *IL6*, Interleukin 6; *PLAC8*, Placenta-specific 8; *SAA1*, Serum Amyloid A1; INHBA, Inhibin Subunit Beta A; *ACTB*, actin beta; GUSB, Glucuronidase Beta.

### Tissue preparation for histopathological examination and immunohistochemical staining

The fixed full-thickness CA samples were dehydrated, embedded in paraffin using routine methods, and sectioned at 5 μM. For histopathological examination, sections were stained with hematoxylin and eosin. The inflammation score was determined based on the amount of inflammation in the placenta and was graded as zero (no inflammation; no inflammatory cells), 1 (mild inflammation; few leukocyte infiltration), 2 (moderate inflammation; moderate leukocyte infiltration) and 3 (severe inflammation; large numbers of inflammatory cells). Average scores for placenta were calculated for each sample based on random evaluation of three different high-power fields. The inflammation score and representative H&E images of the different scores are shown in Figures 1B and C.

For IHC, immunostaining of sectioned tissues was conducted using mouse anti-human calgranulin B (S100A9 1:1000, #MA1-81381 ThermoFisher Scientific), mouse anti-human caspase 7 (CASP7 1:200, #SC-28295, Santa Cruz Biotechnology), mouse anti-human hypoxia inducible Factor 1 subunit alpha (HIF1A 1:200,#MA1-16504, ThermoFisher Scientific), mouse anti-human vascular endothelial growth factor (VEGF 1:50, # MA5-13182, ThermoFisher Scientific), rabbit anti human T-Box Transcription Factor 21 (TBX21 1:400, #PA5-28881, ThermoFisher Scientific), and rabbit anti-human cytochrome P450 family 11 subfamily A member 1 (CYP11A1 1:1000, # NBP1-85368, Novus Biologicals). Paraffin sections were processed with the Leica BOND-MAX system (Leica Microsystems, Buffalo Grove, IL, USA) as described elsewhere [23]. Slides were evaluated at 100 and 200× magnification.

### Statistical analysis

An unpaired T-test was used to evaluate the significance of any changes in gestational age and foal birthweight between control and NP groups. One-way analysis of variance (one-way ANOVA) was used to evaluate the significance of any changes in inflammation score between CRL, NPN, and NPL, followed by a pairwise comparison of means using Tukey’s honestly significant difference (HSD) post hoc test. Differences of *P* < 0.05 were considered as statistically significant and any difference of 0.1 > *P* ≥ 0.05 was considered a trend. Descriptive statistics are expressed as the mean ± standard error of the mean (SEM).

## Results

### Clinical and technical data

The four confirmed nocardioform placentitis (*Amycolatopsis spp.*) cases and four control mares delivered live foals at term (Table 2). The birthweights of foals born to mares with NP were lower (*P* = 0.025). Gestational lengths did not show significant difference (*P* = 0.14) between mares with NP and controls (Table 2). Further details about mares clinical and histopathological findings are presented in Additional file 2. The integrity of RNA recovered from both NP and control tissues was very high (RIN > 9; Table 3).

**Table 2:** **Summary of mare data for tissue collections from nocardioform placentitis and control mares**

Category	Number	Mare age (years)	Gestational age (days)	Foal outcome	Foal birthweight (pounds)	Interval from foaling to tissue collection (minutes)
Nocardioform placentitis	4	11.3 ± 2.4	337 ± 9.3	4 live foals	114 ± 12.3^a^	106 ± 48
Control	4	9.3 ± 0.8	348 ± 6.6	4 live foals	138.8 ± 11.4^b^	38 ± 13

Different superscripts (a and b) indicate significant difference within the same column.

**Table 3:** **RNA integrity of chorioallantois collected from NP and control mares**

Category	RNA integrity number (RIN)
Nocardioform lesion	9.5 ± 0.3
Nocardioform normal	9.7 ± 0.3
Control	9.75 ± 0.26

### Transcriptomic profiling and coverage

**Figure 2:**
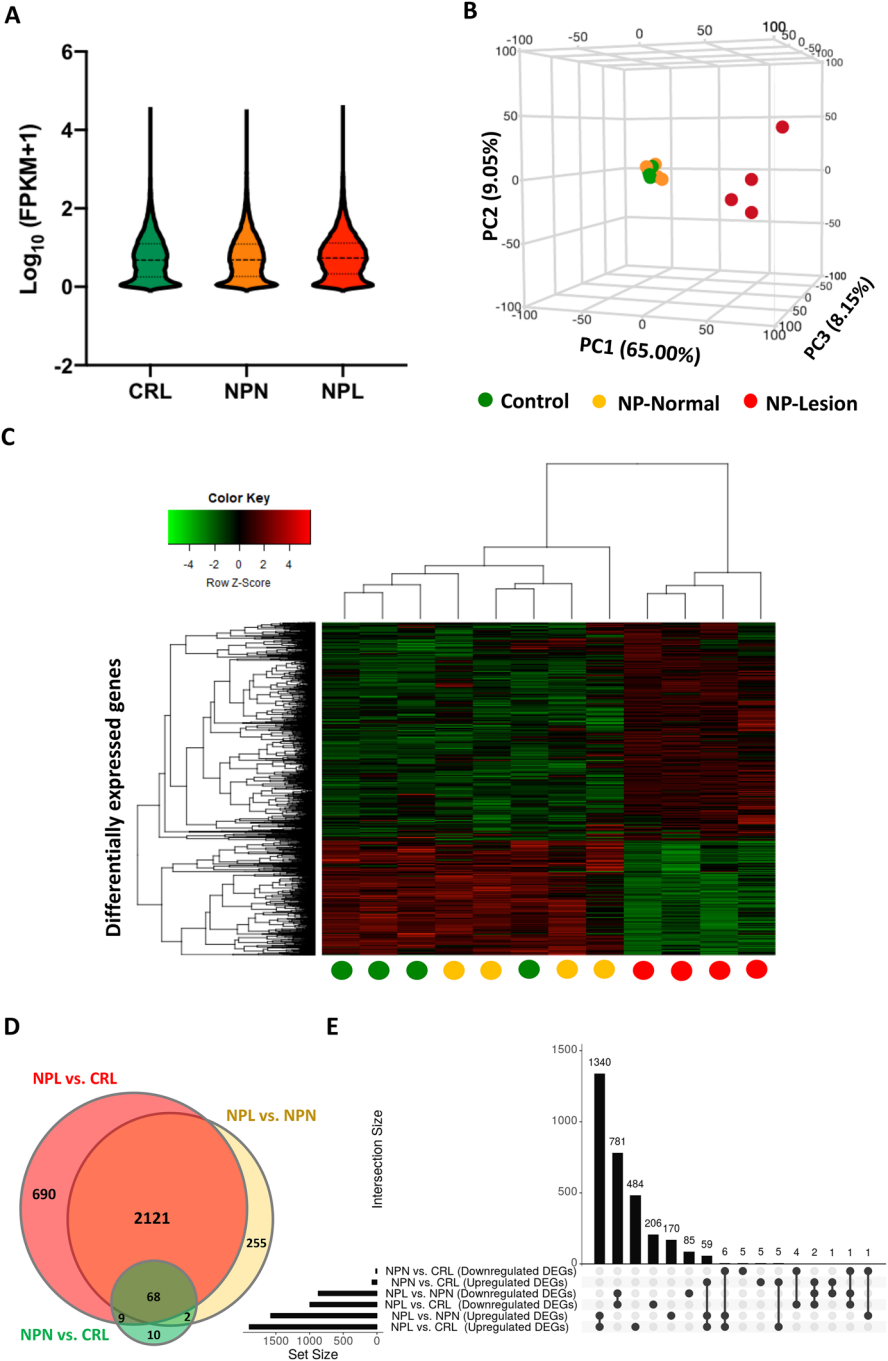
**Transcriptomic Analysis of the Chorioallantois (CA) from Mares with Nocardioform Placentitis.** **A** Violin plot showing transcripts’ FPKM distribution in CA in control (CRL), nocardioform placentitis lesion (NPL), and nocardioform placentitis normal region (NPN) sets. The plots display the distribution of the data using kernel density plots (outer shapes). The inner horizontal lines show the interquartile range (the two outer thin lines), the median (the central thick line). **B** Principal component analysis (PCA) of the equine CA transcriptome from mares in CRL, NPN, and NPL sets. **C** Heat map of differentially expressed genes (DEGs; FDR < 0.05) in NPL vs. CRL, NPL vs. NPN, and NPN vs. CRL sets. The heat map was created using the normalized FPKMs of the DEGs (log10 [FPKM of the DEG + 1]). Normalized expression values are indicated on a color scale with red indicating high expression (positive z-score) and green indicating low expression (negative z-score). The circles at the bottom indicate the sample group (green = CRL, yellow = NPN, and red = NPL). The heat map was generated using the R package “heatmap.2”. **D** Venn diagram representing DEGs in common between NPL vs. CRL, NPL vs. NPN, and NPN vs. CRL sets. **E** UpSet diagram illustrating the intersection between DEGs (upregulated and downregulated) in NPL vs. CRL, NPL vs. NPN, and NPN vs. CRL sets. The nature of each intersection is indicated by the dots below the vertical bar plot. The vertical bars show the number of DEGs in each intersection, while the horizontal bars show the number of DEGs in each comparison. Key; CRL; normal postpartum (control), NPL; nocardioform placentitis lesion, and NPN; nocardioform placentitis normal region.

In total, 94.44 million reads were generated from all cDNA libraries, with an average of 7.87 million reads per sample. An average of 86.9% (range 85.7–88.2%) of reads was mapped to the equine reference genome (EquCab3.0). FPKM distribution and principal component analysis (PCA) of the equine CA transcriptome from mares in CRL, NPN, and NPL is depicted in Figures 2A and B. Further details about individual sample read count, and mapping quality are presented in Additional file 3.

### Differential gene expression

The differentially expressed genes (DEGs) in NPL vs. CRL, NPN vs. CRL and NPL vs. NPN and the overlap between these DEGs are represented in Figures 2C–F and Additional file 4. CA from NPL exhibited differential expression (FDR < 0.05) of 2892 genes (1898 upregulated and 994 downregulated) relative to CRL. Moreover, CA from NPL exhibited differential expression of 2450 genes (1580 upregulated and 870 downregulated) relative to NPN. A total of 2189 DEGs were shared in common between NPL vs. CRL and NPL vs. NPN (Figure 2D). Gene expression of the CA from CRL and NPN clustered together suggesting relatively fewer differences in gene expression (i.e., 89 DEGs) between the two sample sets. This might suggest that NP have a lesser impact on the chorioallantois beyond the lesion zone.

### Gene ontology (GO) enrichment and pathway analysis

**Figure 3:**
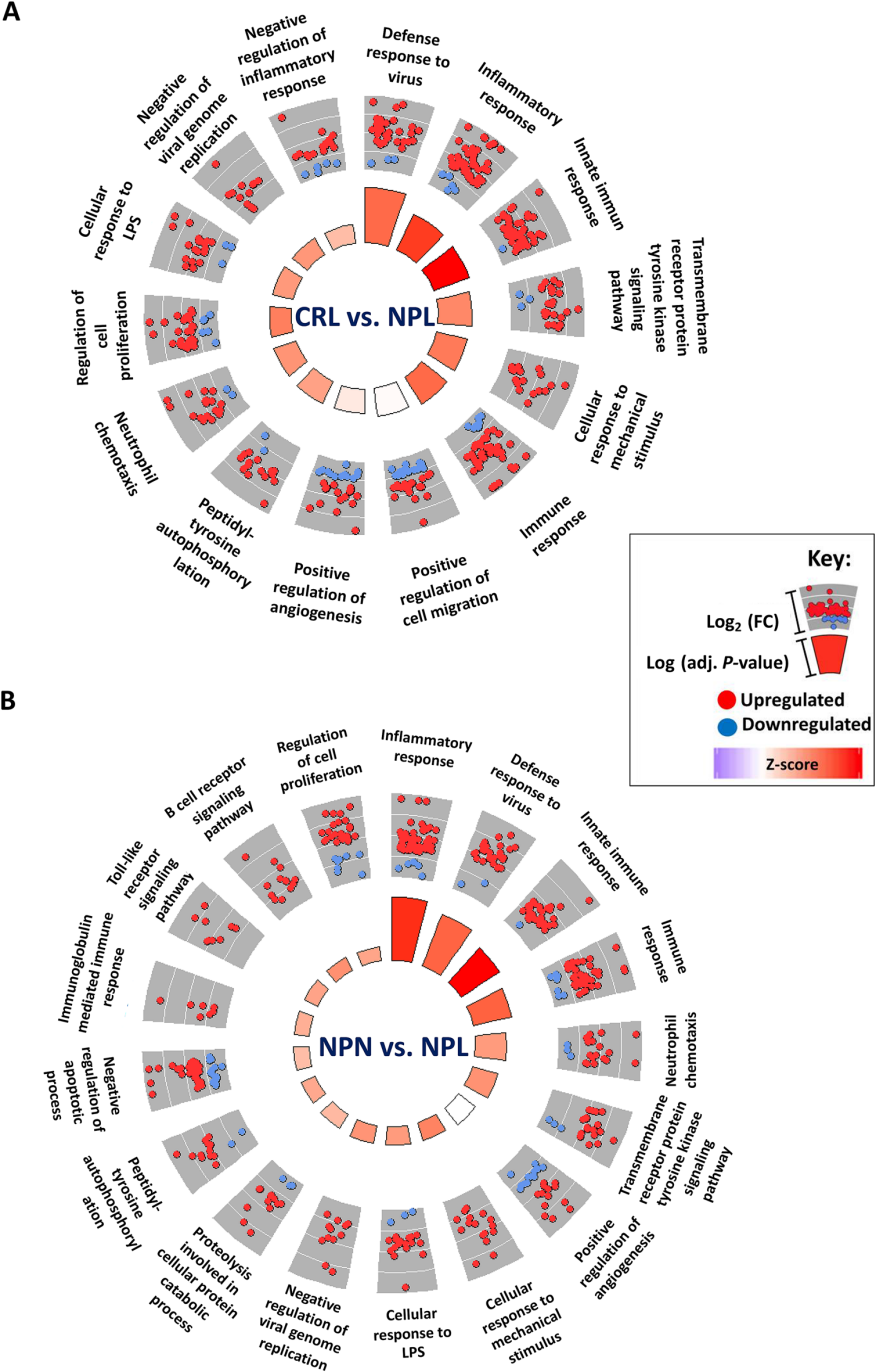
**Overrepresented biological process in equine chorioallantois (CA) in NPL vs. CRL and NPL vs. NPN sets**. Gene ontology (GO) enrichment analysis was used to identify the overrepresented biological process from the DEGs in NPL vs. CRL set (**A**) and NPL vs. NPN set (**B**). The biological processes are visualized by GOplot package in R “GOCircle “. The inner circle is a bar chart where the height of the bar denotes the significance of the GO term (− log10 adjusted P-value), and color links to the z-score. The outer circle displays dot plots of the logFC for the genes in each term. Key; CRL; normal postpartum (control), NPL; nocardioform placentitis lesion, and NPN; nocardioform placentitis normal region.

**Figure 4:**
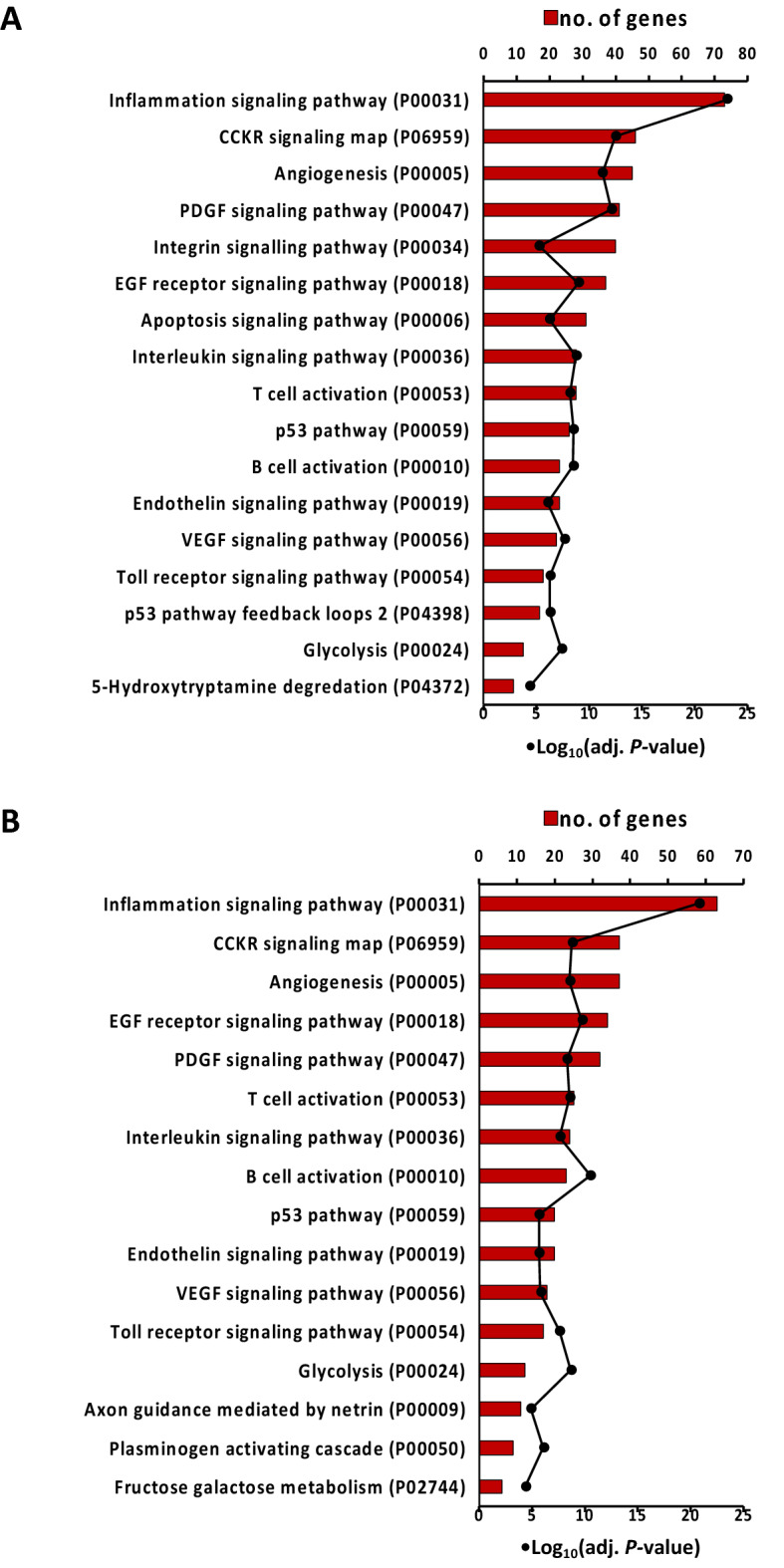
**Overrepresented PANTHER pathways in equine chorioallantois (CA) in NPL vs. CRL and NPL vs. NPN sets.** PANTHER pathway analysis was used to identify the overrepresented pathways from the DEGs in in NPL vs. CRL set (**A**) and NPL vs. NPN set (**B**). The bars represent the number of genes in each overrepresented pathway, and the dotted line represents –Log10 (Adj *P*-value). Key; CRL; normal postpartum (control), NPL; nocardioform placentitis lesion, NPN; nocardioform placentitis normal region, PANTHER; Protein ANalysis THrough Evolutionary Relationships.

Functional annotation of DEGs in NPL demonstrated that these genes were mainly involved in biological processes associated with inflammatory response, immune response, immunoglobulin mediated immune response, cellular response to lipopolysaccharide, toll-like receptor signaling pathway, B cell receptor signaling pathway, defense response to virus, angiogenesis, and chemotaxis, among others (Figures 3A and B). Moreover, pathway analysis of DEGs in NPL revealed that these genes are involved in relevant pathways such as inflammatory signaling (interleukin signaling, inflammation mediated by chemokine and cytokine signaling pathway, toll-like receptor signaling, B cell activation, and T cell activation), angiogenesis-related pathways (VEGF signaling, angiogenesis, endothelin signaling pathway and PDGF signaling pathway), cytostructural integrity (integrin signaling pathway), and apoptosis signaling pathway, among others (Figures 4A and B).

### Upstream regulators and their targets

**Figure 5:**
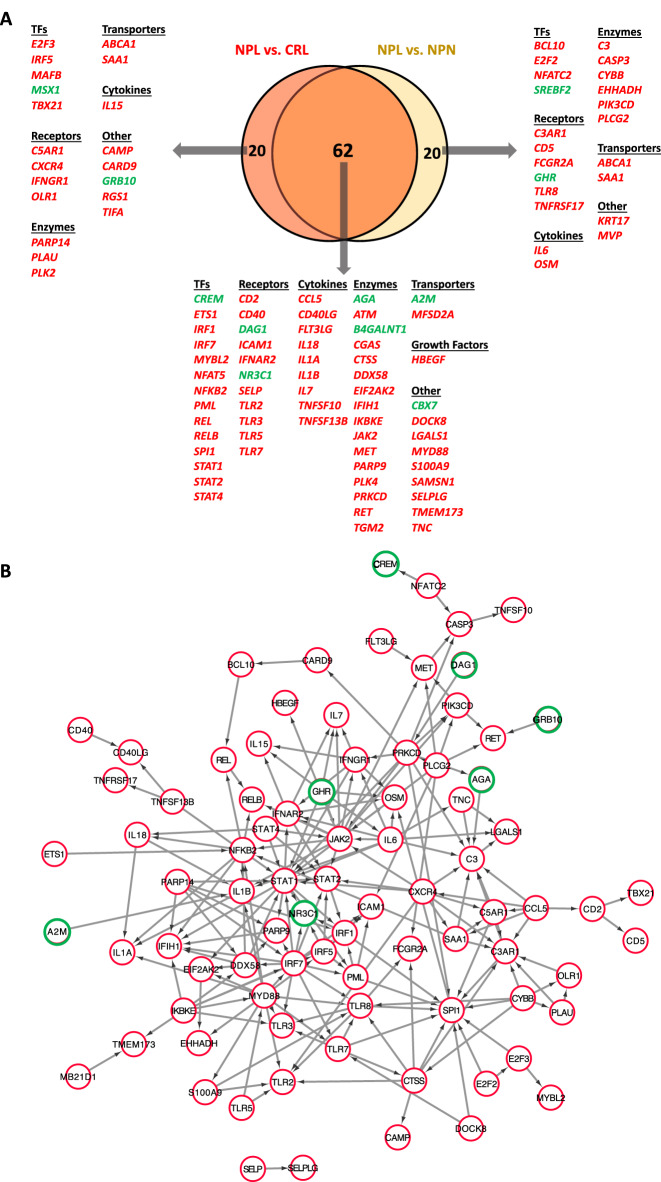
**Potential upstream regulators in equine chorioallantois (CA) during nocardioform placentitis (NP) as identified by upstream regulator analysis performed on Ingenuity Pathway Analysis (IPA).** **A** Venn diagram representing potential upstream regulators in common between NPL vs. CRL and NPL vs. NPN sets. **B** Protein–protein interactions network between upstream regulators identified NPL vs. CRL and NPL vs. NPN sets. Protein–protein interactions network was generated by STRING version 10.3. and visualized with Cytoscape 2.8.6. Red circles indicate activated upstream regulator, while green circle indicate inhibited upstream regulator. Key; CRL; normal postpartum (control), NPL; nocardioform placentitis lesion, and NPN; nocardioform placentitis normal region.

The upstream regulator analysis identified 73 activated upstream regulators (*P*-value of overlap is < 0.05 and Z-score ≥ 2) among the DEGs in both NPL vs. CRL and NPL vs. NPN (Figure 5A). Those activated upstream regulators included 55 regulators in common between both sets. Those 55 regulators include genes coding for transcription factors (*ETS1*, *IRF1*, *IRF7*, *MYBL2*, *NFAT5*, *NFKB2*, *PML*, *REL*, *RELB*, *SPL1*, *STAT1*, *STAT2* and *STAT4*), transmembrane receptors (*CD2*, *CD40*, *ICAM1*, *SELP*, *TLR2*, *TLR3*, *TLR5*, and *TLR7*), and cytokines (*CCL5*, *CD40LG*, *FLT3LG*, *IL1A*, *IL1B*, *IL7*, *IL18*, *TNFSF10*, *TNFSF13B*), among others. Also, this analysis identified nine inhibited upstream regulators (*P*-value of overlap is < 0.05 and Z-score ≤ -2) among the DEGs in both NPL vs. CRL and NPL vs. NPN. Those inhibited upstream regulators included seven regulators (*A2M, AGA*, *B4GALNT1*, *CBX7*, *CREM*, *DAG1*, and *NR3C1*) in common between both sets. The identified upstream regulators and their target molecules in NPL vs. CRL and NPL vs. NPN are represented in Additional files 5A and B, respectively. The overlap between identified potential upstream regulators in NPL vs. CRL and NPL vs. NPN is depicted in Figure 5A. Moreover, the interaction between identified potential upstream regulators is illustrated in Figure 5B.

### Weighted co-expression network analysis (WGCNA)

**Figure 6:**
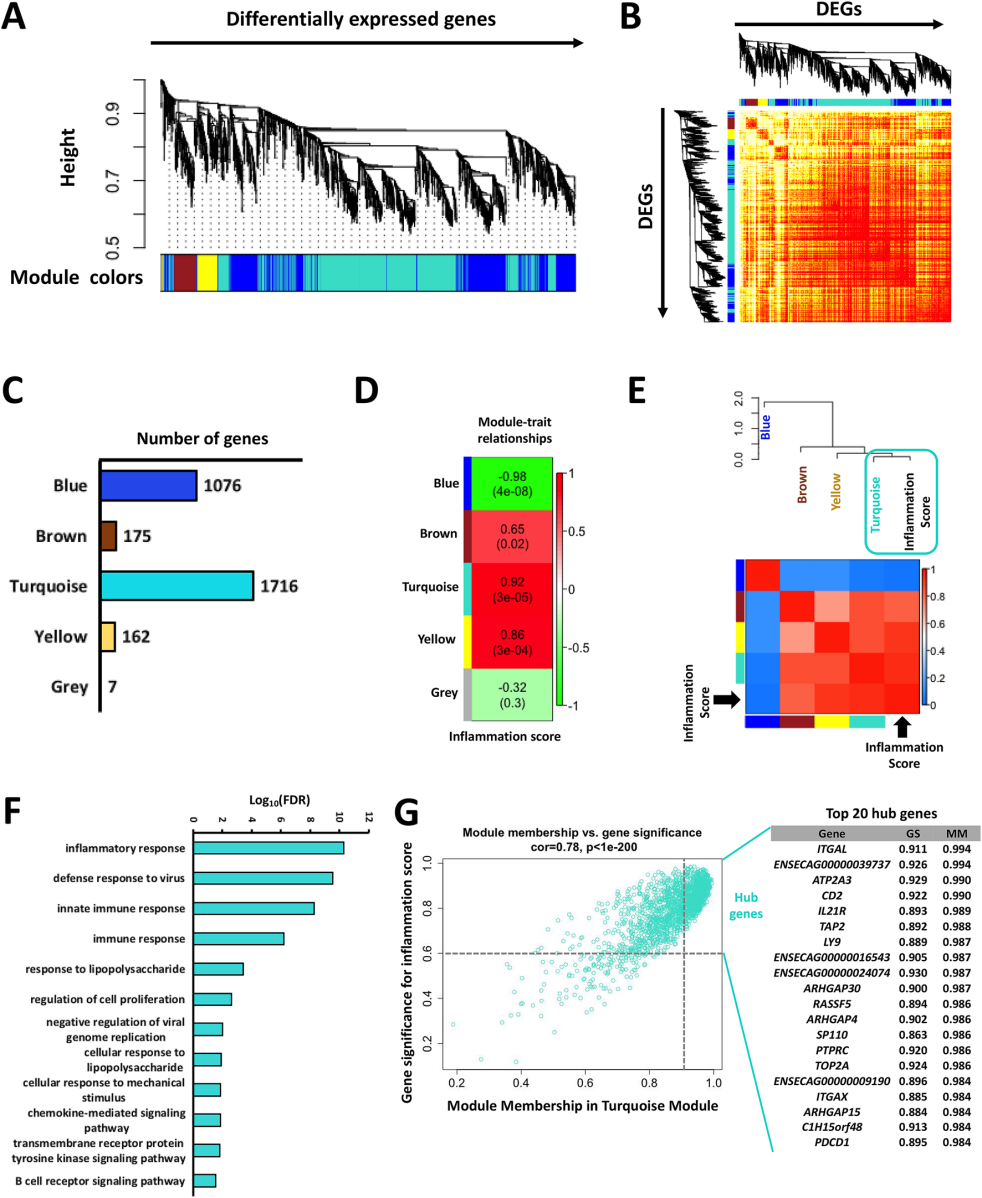
**Weighted gene expression co-expression network analysis (WGCNA) of DEGs in equine chorioallantois (CA) during nocardioform placentitis dataset**. **A** Gene module (clusters) identification as determined by WGCNA. **B** Matrix with the module-trait relationships and corresponding p-values of the modules on the y-axis and selected traits related with treatment on the x-axis. The y-axis is colored according to the correlation, with red representing a strong positive correlation and green representing a strong negative correlation. **C** Number of genes per module. **D**, **E** Module-trait correlation heatmap based on correlation analysis of module eigengenes (MEs) and inflammation score in placenta (*n* = 12). The turquoise, yellow, and brown modules presented a significant positive correlation with inflammation score (*p*-values < 0.05). **F** Over-represented biological process in the turquoise module. **G** A scatter plot of GS for inflammation score versus the MM in the turquoise module.

WGCNA was carried out to gain further insights on the DEGs co-expression patterns and to identify the genes with the highest interaction or connectivity (hub genes) among those DEGs in NPL vs. CRL and NPL vs. NPN. Co-expression analysis of the DEGs in NPL identified five gene modules (clusters) as presented in Figure 6 and Additional file 6. Among these modules, turquoise, yellow and brown modules were positively associated with inflammation score. But the blue module was negatively correlated to this trait. It is worth noting that, the turquoise module showed the highest adjacency to inflammation score trait as presented in Figure 6E.

### Ligand-receptor interaction analysis

**Figure 7:**
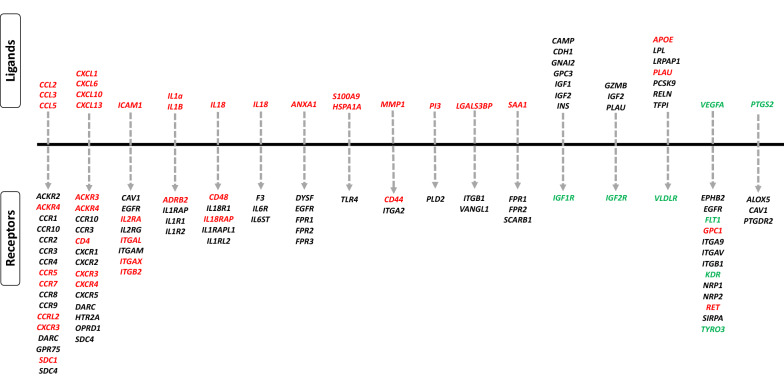
**Ligand-receptors interaction analysis of DEGs during nocardioform placentitis.** Genes shown in red are significantly upregulated in the chorioallantois (CA) from the nocardioform placentitis lesion (NPL) in comparison to normal postpartum (CRL). Genes shown in green are significantly downregulated in NPL in comparison to CRL. Genes shown in black are those that did not show any significant change in NPL in comparison to CRL.

To gain further insights on the interaction between DEGs, ligand-receptor interaction analysis identified 1038 possible interactions in NP. These interactions elucidate the impact of NP on placental autocrine and paracrine signaling in comparison to normal parturition. A representative set of Ligand-receptor interactions is presented in Figure 7 and the complete list of interactions is provided in Additional file 7.

### RT-qPCR validation

**Figure 8:**
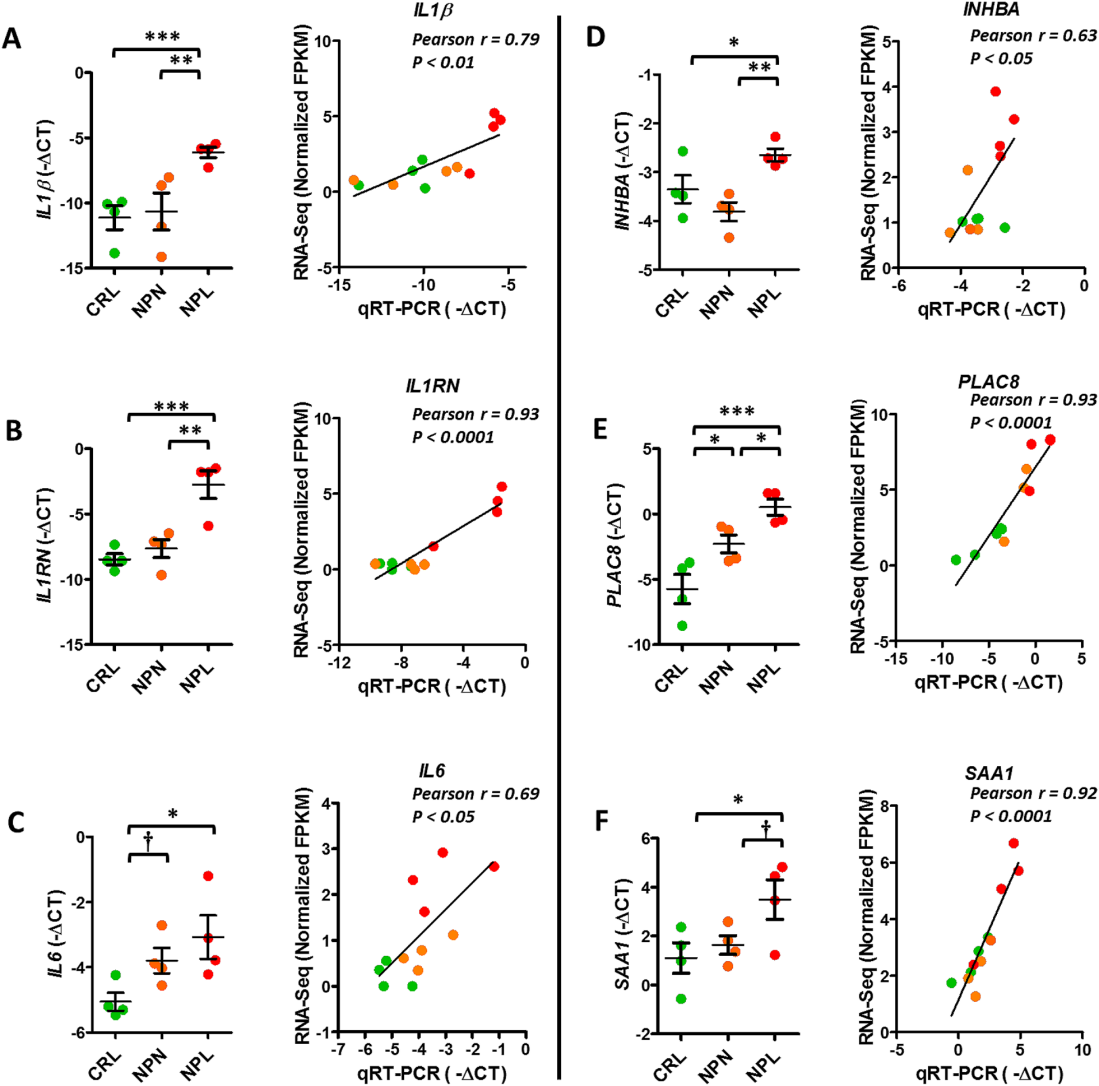
**RT-qPCR validation of six selected differentially expressed genes from RNA-sequencing results**. Expression of each mRNA was determined by qRT-PCR, normalized to *ACTB* and *GUSB*, expressed as −∆CT. Data are presented as a dot plot, and the middle horizontal line represents the mean while error bars represent the standard error of the mean (SEM). Asterisks indicate the presence of a significant difference between groups (*P < 0.05; **P < 0.01, *** *P* < 0.001). Dagger (†) indicates the presence of a trend (0.1 > P ≥ 0.05). Pearson's correlation coefficient (r) between mRNA expression results from RT-qPCR (−ΔCT) and the RNA-sequencing (FPKM) is represented for each transcript. Key; *IL1β*, Interleukin 1β; *IL1RN*, Interleukin 1 Receptor Antagonist; *IL6*, Interleukin 6; *PLAC8*, Placenta-specific 8; *SAA1*, Serum Amyloid A1; *INHBA*, Inhibin Subunit Beta A; *ACTB*, actin beta; *GUSB*, Glucuronidase Beta.

RT-qPCR confirmed the differential expression of six genes in NPL in comparison to CRL and/or NPN group as illustrated in Figure 8. Analysis of the correlation between gene expression results from RT-qPCR (−ΔCT) and the RNA-sequencing (normalized FPKM) showed that all genes had a significant correlation between the two methods (Figure 8).

### Immunohistochemistry

**Figure 9:**
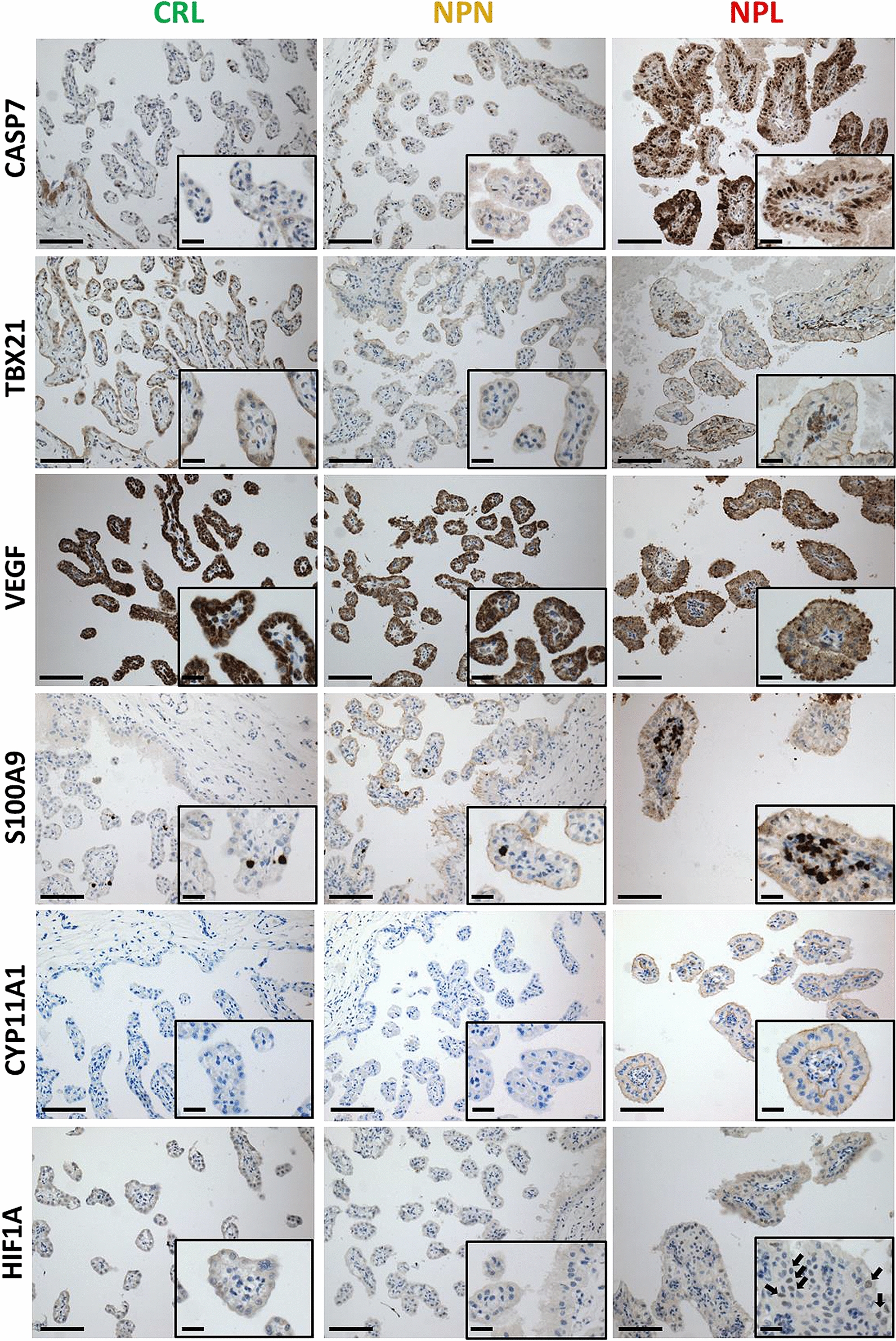
**Representative photomicrographs of the equine placenta retrieved from CRL, NPN, NPL, and immunostained for S100A9, CASP7, HIF1A, VEGF, TBX21, and CYP11A1.** Bar = 100 µm. Key; CRL; normal postpartum (control), NPL; nocardioform placentitis lesion, and NPN; nocardioform placentitis normal region, CASP7; caspase 7, HIF1A; hypoxia inducible Factor 1 subunit alpha, VEGF; vascular endothelial growth factor, CYP11A1; cytochrome P450 family 11 subfamily A member 1.

The protein localization and staining intensity for CASP7, TBX21, VEGF, S100A9, CYP11A1, HIF1A, and SAA1 in equine CA retrieved from CRL, NPN, and NPL sets are shown in Figure 9. Based upon immunolocalization, CASP7 was predominantly expressed in the nucleus and cytoplasm of chorionic epithelium in NPL compared to CRL and NPN. S100A9 and TBX21 appeared to be mainly expressed in immune cells in CA retrieved from CRL, NPN, and NPL with a higher number of S100A9 and TBX21 positive cells in NPL. The expression of both VEGF and CYP11A1 was predominant in the cytoplasm of the chorionic epithelium. VEGF showed a higher immunolabeling intensity in CA retrieved from CRL and NPN compared to NPL. In contrast, CYP11A1 showed a higher immunolabeling intensity in CA retrieved from NPL compared to CRL and NPN. HIF1A was mainly expressed in the nucleus of chorionic epithelium, endothelial cells and vascular smooth muscle cells in blood vessels as well as scattered stromal cells within the CA with a higher number of HIF1A positive cells in NPL set. It is worth noting that inflammatory cells did not display any positive immunolabeling for HIF1A. Overall, the immunostaining intensity for all evaluated targets was consistent with the transcript expression in the current RNA-Seq dataset.

## Discussion

To the authors’ knowledge, this is the first study to assess transcriptomic changes in the CA of mares with spontaneous NP (*Amycolatopsis *spp*.*). The current study presents a distinct transcriptome signature of equine NP (*Amycolatopsis *spp.) and adds to the understanding of the key regulators and molecular mechanisms involved in the disease. Overall, a large number of genes were differentially expressed between NP-lesion and control mares, which is similar to studies on equine ascending placentitis [24–26] and human intra-amniotic infections [27]. Our results revealed that NP is dominated by Toll-like receptor signaling, inflammasome activation, inflammatory signaling and apoptosis. Although, the current study focused on the characterization of NP transcriptome associated with *Amycolatopsis spp*, which is the most common bacteria associated with this disease, further studies are still warranted to assess the transcriptome of NP associated with other bacteria (e.g., *Crossiella equi*, *Streptomyces atriruber* and *Streptomyces silaceus*).

Although, the NPN samples didn’t show significant difference in macroscopic and microscopic appearance (i.e., inflammation score) compared to CRL samples, we identified 89 DEGs in NPN vs. CRL set. This transcriptomic difference could be attributed to exocrine molecules released from the lesion (NPL) and consequently affect the transcriptome of unaffected tissue (NPN). This notion is supported by two recent retrospective studies which have described the serum profile of mares with focal mucoid placentitis (*n* = 6; two placentas were PCR positive for the *Amycolatopsis* ssp, while the other four had no bacteria detected) and found alterations in endocrine, cytokine, and feto-secretory markers in the weekly assessed samples [28, 29]. In brief, these showed an increase in IL-2, IL-5, IL-6, IL-10 and TNF in the maternal serum. Interestingly, the present study revealed that IL-6 is a potential upstream regulator for the DEGs identified in NPL vs. NPN dataset, which again supports our notion. Additionally, the relatively small number of DEGs in the NPN vs. CRL set was reflected in the close clustering of NPN and CRL samples (low inflammation score) far from NPL samples (high inflammation score) in the PCA and the heatmap. Noteworthy, the present study identified 2189 DEGs (represent 69.5% of DEGs) in common between NPL vs. CRL and NPL vs. NPN sets. These DEGs are the main focus of this discussion section unless otherwise stated.

The central role of inflammatory mediators during placental infections is well established in women, mice and mares [12, 30–32]. Likewise, the current study demonstrated that equine NP is principally dominated by inflammatory and immune responses, as revealed by functional genomics analysis of DEGs. In brief, the NP transcriptome revealed the upregulation of a set of genes encoding the key regulators of the inflammatory cascade. These include pattern recognition receptors (PRRs) such as Toll-like receptors (*TLR*s) and Nod-like receptors (*NLR*s). Of note *TLRs* are the primary and earliest recognition mechanism for pathogen associated molecular patterns (PAMPs) unique to the microorganisms with subsequent activation of the inflammatory cascade [33, 34]. In the present study, several TLRs (*TLR1*, *TLR2*, *TLR3*, *TLR5*, *TLR7* and *TLR8*) were significantly upregulated in NP. It is noteworthy that TLR1/TLR2 heterodimers are responsible for recognition of gram-positive bacteria, consistent with the *Amycolatopsis spp.* infection. Meanwhile, TLR5 is responsible for recognition of bacterial flagellin [33]. On the other hand, TLR3, TLR7 and TLR8 are responsible for recognition of viruses [33]. The upregulation of the later TLRs might suggest presence of a viral component accompanying NP and/or indicate the upregulation of ligands that could activate these receptors [33]. This notion was somewhat supported by the overexpression of viral-related pathways (defense response to virus and negative regulation of viral genome replication) in NPL as indicated by gene ontology analysis. Upstream regulator analysis revealed the central role of *TLRs* (*2*, *3*, *5*, *7*, and *8*) in NP. Furthermore, *TLR2*, *TLR7*, and *TLR8* showed up as hub genes in the turquoise module in the WGCNA database. Taking all together, these findings address the principal role of *TLRs* as key regulators triggering the inflammatory cascade associated with NP. Strategies to block TLRs hold potential for therapies to block the inflammatory cascade and to forestall NP. This notion is supported by studies showing that TLR antagonists (TLRAs) were highly effective in preventing preterm birth induced by lipopolysaccharides (LPS), heat-killed E. coli or platelet activating factor (PAF) in primates and mice [35, 36].

*NLR* signaling is a critical component in inflammasome activation [37]. Inflammasomes are cytosolic multi-protein complexes that orchestrate the inflammatory cascade [38]. Recently, we have elucidated that inflammasome activation is implicated in equine ascending placentitis [24]. Similarly, several inflammasome-related transcripts (*NLRC4*, *NLRC5*, *CASP1*, *IL1B*, *IL18*, and *GSDMD*) were significantly upregulated during NP in the current study. Moreover, *NLRC4*, *NLRC5*, *IL1B*, *IL18*, and *GSDMD* showed up as hub genes in the turquoise module in the WGCNA database. Furthermore, *IL1B* and *IL18* showed up as upstream regulators in the present study. Taken all together, these findings address the crucial role of *NLRs* in triggering the inflammatory response associated with NP. Therefore, targeting NLRs along with TLRs during NP might be promising for blocking the inflammatory cascade and to forestall placentitis-induced preterm birth. This notion is supported by recent studies showing that the treatment of intra-amniotic infection using NLR inflammasome inhibitors [39] resulted in inhibition of prostaglandin synthesis with subsequent prevention of infection-associated preterm birth in mice.

Among the mediators of inflammatory pathways, chemokines are crucial for the activation and recruitment of immune cells to the infection site [34, 40–43]. The infiltrating immune cells are implicated in local inflammation, partly by releasing cytokine and matrix metalloproteinases (MMPs). These molecular events have been reported in human chorioamnionitis and equine ascending placentitis [24, 34, 40–43]. Similarly, in the present study, members of these gene groups of chemokines (e.g., *CCL2*, *CCL8*, *CCL5*, *CXCL6*, and *S100A9*), cytokines (e.g., *IL1α*, *IL1β*, *IL6*, *IL7*, *IL15* and *IL18*), and MMPs (e.g., *MMP1, MMP3*, *MMP8*, and *MMP12*) were upregulated during NP. The upregulation of MMPs is implicated in extracellular matrix (ECM) degradation and apoptosis [24, 44–48]. Likewise, the apoptosis signaling pathway and several apoptosis-related genes (*CASP3*, *CASP4*, *CASP7*, *CASP8*, and *CASP10*) were overexpressed during NP. The implication of MMPs and apoptosis in placental separation have been described during term and preterm labor in women, mares and ruminants [24, 44–48]. Among the previously discussed molecules, *IL1α*, *IL1β*, *IL6*, *IL7*, *IL18*, *CCL5*, *S100A9*, and *CASP3* showed up as upstream regulators for NP. Moreover, *CCL* (*2*, *3*, *7*, and *8*), *CXCL* (*6*, *8*, and *10*), *IL* (*1α*, *1β*, *6*, *7*, *18*), *CASP* (3, 4, 7, 8, and 10), *S100A9* and *MMP8* showed up as hub genes in NP as elucidated by WGCNA. Additionally, our results revealed that the infiltrating immune cells significantly affected the transcriptomic signature of nocardioform placentitis. For instance, the inflammation score trait (i.e., degree of immune cell infiltration) showed the highest adjacency to the turquoise module, which is the largest module (includes 54.71% of DEGs) in WGCNA dataset. Therefore, the DEGs in this module might be triggered by the infiltration of the placenta by immune cells. In support, pathway analysis of these DEGs revealed that they are involved in pathways related to inflammatory cascade and immune response. Taken together, these findings highlight the central role of immune cells in orchestrating the localized placental inflammation and consequent placental separation during NP.

Although the recruitment of immune cells to the infection site is a principal element of the innate immune response against the invading pathogenic microorganism, host defense peptides (HDPs), also known as antimicrobial peptides (AMPs), represent another vital element in this immune response [49–51]. HDPs represent potent and broad-spectrum antimicrobial properties. The expression of genes coding for HDPs is induced by transcription factors due to the activation of *TLRs* [49–51]. We recently reported that equine ascending placentitis is associated with upregulation of several genes coding for HDPs [24]. In agreement, NP was associated with upregulation of several HDPs such as lysozyme (*LYZ)*, peptidase inhibitor 3 (*PI3*), secretory phospholipase A2 (*sPLA2*; *PLA2G2A*), myeloid cathelicidin 3 (*ECATH3*), granulysin (*GNLY*), secretory leukocyte peptidase inhibitor (*SLPI*), acid phosphatase 5, tartrate resistant (*ACP5*), and psoriasis (*S100A7*). In line with our findings, the upregulation of several HDPs has been reported in women with intra-amniotic infection [52, 53] and mares with ascending placentitis [24]. Additionally, several reports have described the impressive in vivo activities of HDPs [54, 55]. As a result, there has been growing interest in HDPs as new treatment strategies for bacterial infections [54, 55]. So far, the efficacy of HDPs as a potential treatment for equine NP has not been tested.

**Figure 10:**
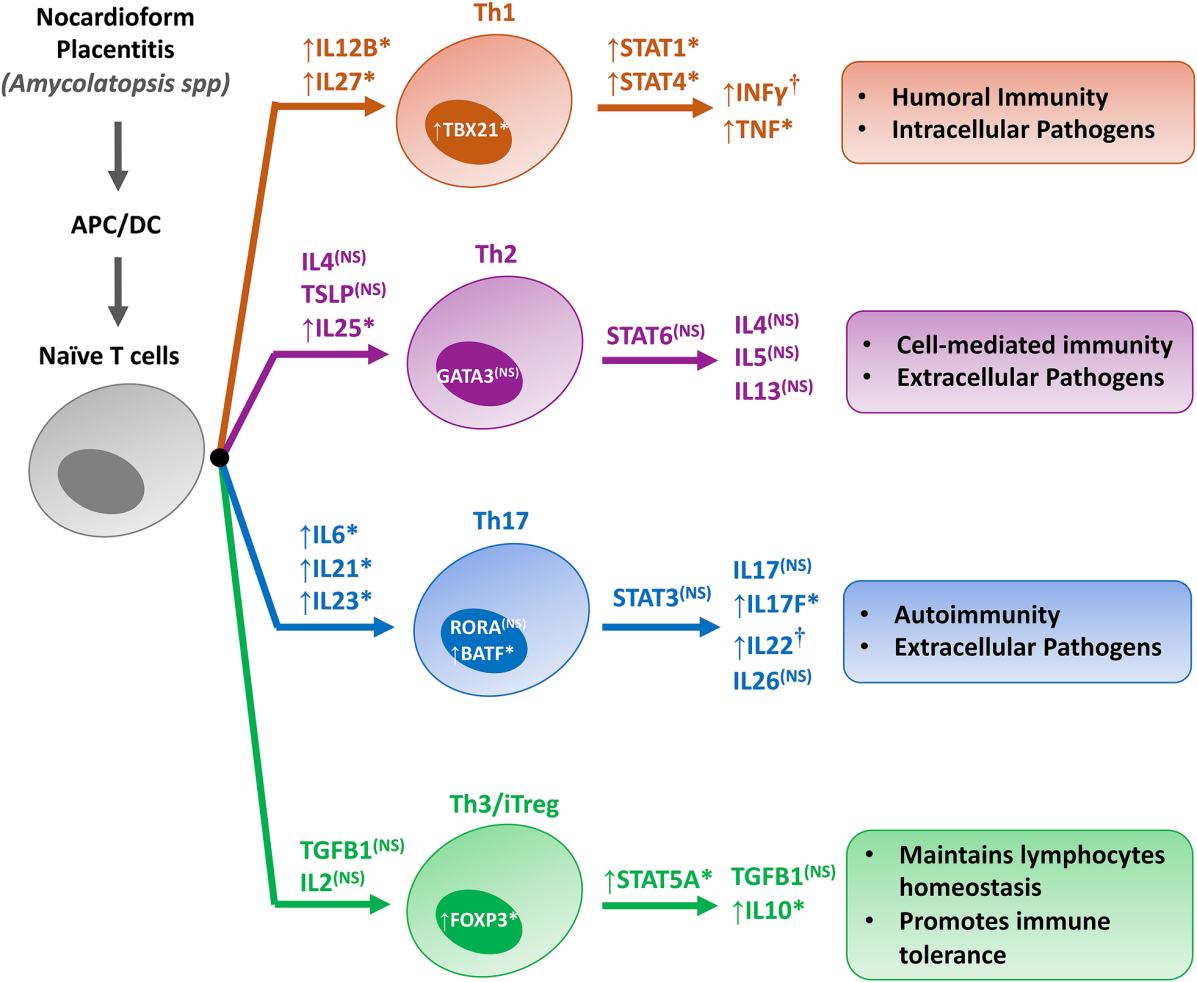
**Schematic illustration elucidating the expression pattern of T. cells related transcripts during NPL in comparison to CRL**. The FPKM of the selected genes were extracted from the transcriptome dataset and analyzed using student T-test. The arrow directions indicate if the transcript is upregulated or downregulated in NPL in comparison to CRL. Key; CRL; normal postpartum (control), NPL; nocardioform placentitis lesion, TBX21; T-Box Transcription Factor 21, IL; Interleukin, STAT; signal transducer and activator of transcription.

In addition to the activation of the innate immune response, NP also appears to lead to an upregulation of the adaptive immune system, including T cell lymphocyte development and proliferation. T cell lymphocytes are believed to be crucial for pregnancy maintenance and proper embryo development, in addition to antigen-specific defense against pathogen [56, 57]. While Th1 and Th2 cell types are essential for defense against intracellular and extracellular pathogens respectively, it is the balance between Th17 and Treg response which allows the body to carry the pregnancy to term [24]. A disruption of this balance has been indicated in various pregnancy-related complications, including amniotic and placental infection [58–60] and recurrent unexplained abortion [61–64]. In the present study, NP was associated with an increase in factors relating to the Th1 response (*STAT1*, *STAT4*, *TBX21*, *IL15*, *IL18*). An upregulation of various Th17-related transcripts was associated with NP (*BATF*, *IL17RC*, *IL21R*, *S100A9*), but this was alongside a similar increase in Treg-related transcripts (*CXCR4*, *SELL*, *IL2RA*, *IL10RA*). An increasing Th17 response along with a dysregulated Treg response has been noted in various types of pregnancy-related complications [57, 59, 63, 65, 66], and yet both apparently increased in NP. This dual increase may explain the response to the pathogen and the chronic nature of this disease, as all affected fetuses resulted in live birth. The expression pattern of T-cells related transcripts in NPL vs. CRL is summarized in Figure 10.

In the current study, placental inflammation was associated with aberrant expression of several placenta-regulatory genes. For example, placenta-specific 8 (*PLAC8*), and galectin 1 (*LGALS1*) were significantly upregulated during NP, while placenta-specific ATP-Binding Cassette Transporter (*ABCG2*), chorion-specific transcription factor GCMa (*GCM1*), endothelial PAS domain protein 1 (*EPAS1*), and nuclear receptor subfamily 3 group C member 1 (*NR3C1*) were significantly downregulated during the disease. Similarly, we recently reported that equine ascending placentitis is accompanied by abnormal expression of *PLAC8*, *LGALS1*, *ABCG2*, *GCM1*, and *NR3C1* [24, 67]. It is likely that the altered expression of these placenta-regulatory genes will adversely affect the placental functions during placentitis.

Within the NP transcriptome signature, we identified aberrant expression of genes in insulin-like growth factor (IGF) signaling: *IGFBP2*, *IGFBP6*, *IGFR1*, and *IGFR2*. The upregulation of the *IGFBP2* and *IGFBP6* could have multiple paracrine and autocrine consequences throughout the context of NP. Secreted IGFBP2 and IGFBP6 have a high affinity for IGF1 and IGF2, respectively, reducing their bioavailability to activate IGF signaling [68]. Similarly, we have recently reported that equine ascending placentitis is associated with upregulation of *IGFBP2*, *IGFBP4*, and *IGFBP7*, and downregulation of *IGFR1* [24]. It is noteworthy that placental IGF signaling is implicated in fetal growth [69, 70]. Therefore, the upregulation of *IGFBP2* and *IGFBP6* and downregulation of *IGFR1* and *IGFR2* during NP may suggest a reduction in IGF signaling, consistent with the lower birthweight we reported in the current study. Additionally, the present study revealed altered expression of several solute carrier (*SLCs*) genes. Of note, SLC is a family of membrane-bound proteins (more than 300 proteins) that facilitate the transport of a wide range of substrates across biological membranes, including the placenta [71]. Our results revealed downregulation of approximately 31 *SLCs*, including glucose transporters (*SLC2A1*, *SLC2A10*, and *SLC2A12*), amino acids transporters (*SLC7A1*, and *SLC38A4*), fatty acids transporters (*FABP1*, *FABP3*, *VLDLR SLC25A17*, *SLC27A1* and *SLC25A17*), vitamin transporters (*SLC19A3*), and adenosine triphosphate (ATP) transporter (*SLC25A17*), among others. The downregulation of the above-mentioned *SLCs* could result in a decline in transportation of maternal nutrients to the growing fetus. Again, these findings are consistent with lower birthweight of foals in the current study. Moreover, our results showed that NP was associated with dysregulation of angiogenesis, as reflected by the downregulation of angiogenesis-related genes (e.g., *VEGFA*, *KDR*, *ANGPT2* and *FLT1*). Angiogenesis dysregulation could result in a decline in placental blood perfusion (ischemia) and, consequently, hypoxia in placental tissues [72]. This notion is supported by the upregulation of hypoxia-related genes (*HIF1A* and *EGLN3*) in NPL. Therefore, the use of medicaments that improve placental angiogenesis and/or blood flow might be beneficial for NP treatment. Overall, the previously discussed molecular mechanisms underlying dysregulation of placental angiogenesis and nutrient transportation, as well as placental hypoxia, could explain placental insufficiency during NP.

Identifying the gene expression signature that is intrinsic for equine NP is potential for future in vitro and/or in vivo studies. Additionally, if the proteins coded by these genes are released from the placentitis lesion into the maternal circulation and can be measured, these proteins might serve as reliable and sensitive biomarkers for equine NP. The current study revealed that 105 genes were exclusively expressed in NPL in comparison to control (i.e., zero FPKM in CRL set). Some of these transcripts encode proteins that have been proposed as potential biomarkers for intra-amniotic infection or inflammation in women with preterm labor [73, 74], such as *ENSECAG00000008686* (psoriasis; *S100A7*) and *ENSECAG00000010615* (calgranulin B; *S100A9*). On the other hand, the top ten exclusively expressed genes (based on abundance in NPL) were novel genes. Gene homology analysis of these ten genes revealed that six of them are homologous to human and mouse immunoglobulin kappa constant (IGKCs; *ENSECAG00000001923* and *ENSECAG00000001923*), immunoglobulin lambda constant (IGLCs; *ENSECAG00000015109*, *ENSECAG00000031522*, and *ENSECAG00000039599*), and immunoglobulin heavy variable (IGHVs; ENSECAG00000028730), while the remaining four genes did not show any homology to human and mouse genes. Further studies are required to assess the utility of the immunoglobulins as potential biomarkers for equine NP. Overall, these NP exclusively expressed transcripts represent an intrinsic signature for equine NP, and further studies are required to elucidate their role in the NP pathway. Further studies to assess their associate proteins as potential biomarkers for equine NP are warranted.

In the present study, the RNA integrity of RNA isolated from CA collected within three hours after foaling was consistently high (RIN > 9) which suggests that this sampling strategy of collecting CA on farm and preservation in RNA*later*® could be effective for further studies on term equine placenta. This observation is similar to studies of human term placenta where preservation of decidua in RNA later yielded high RNA integrity [75].

**Figure 11:**
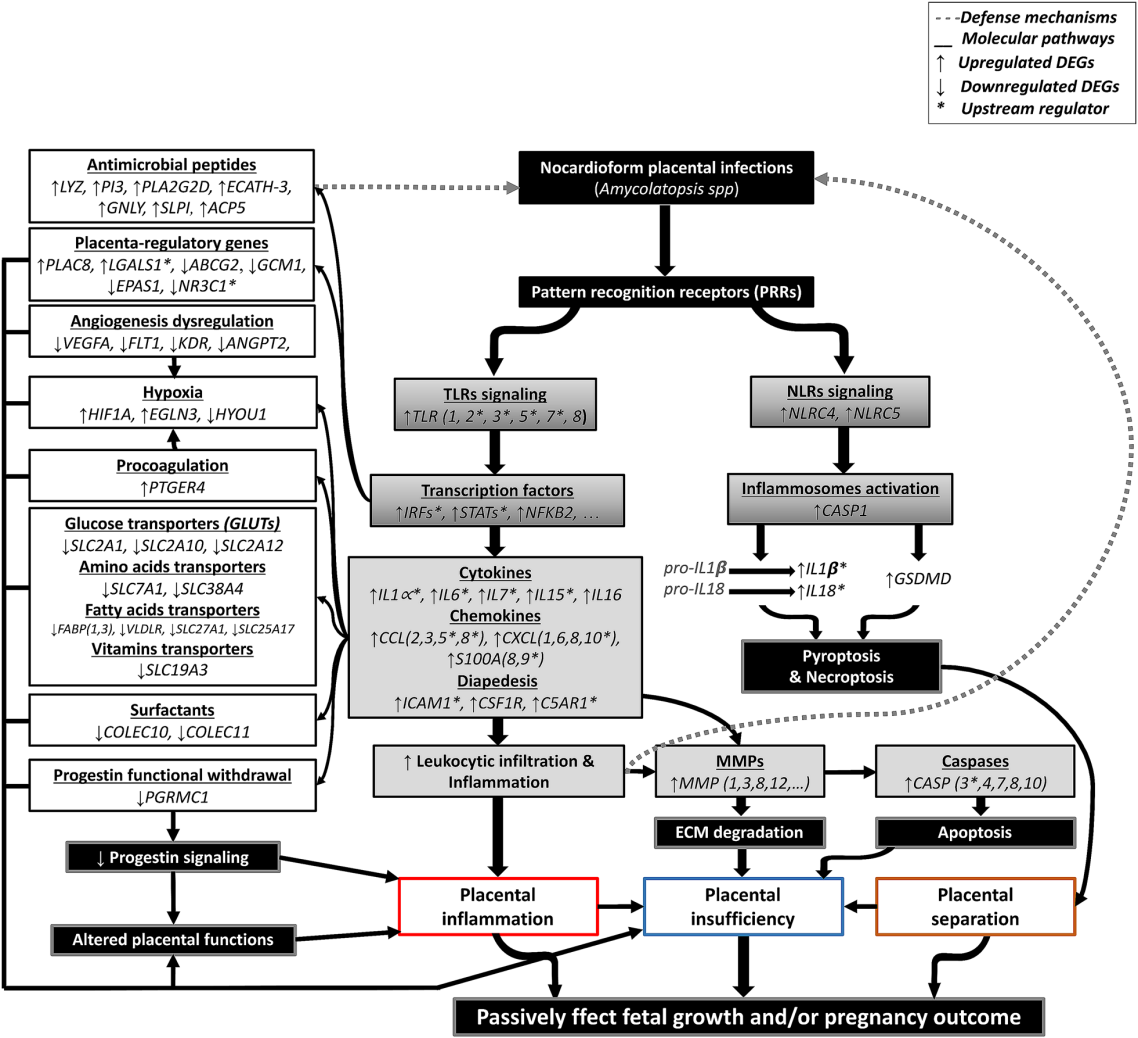
**Schematic illustration explaining the possible mechanisms associated with equine nocardioform placentitis in light of the current study.** The arrow directions indicate if the gene is upregulated or downregulated in the current study.

There are some potential limitations to our study. These limitations relate to obtaining samples at the beginning of the disease because nocardioform placentitis is always diagnosed in the disease’s late stages, and it is difficult to obtain prepartum samples from clinical cases. Moreover, there is no valid experimental model for the disease that would us to collect samples at the early stages of the disease. Similar to these limitations, human studies that are concerned with clinical chorioamnionitis or spontaneous preterm birth use a similar sampling time point (i.e., no samples are obtained at the beginning of the disease), and results from these studies are still acknowledged in human reproductive science [34, 40]. Furthermore, it is believed that the bacterial infection during NP starts at the center of the lesion and then progresses/expands outward. Therefore, we believe that the current sampling site (i.e., the margin of the lesion [NPL]) would reflect many of the molecular mechanisms associated with the early interaction between the bacteria and the chorioallantois. In conclusion, on-farm collection of the CA from mares within three hours after foaling yielded RNA suitable for analysis by high-throughput sequencing. This is the first study to describe the equine CA transcriptome during NP. Our results revealed the central role of *TLRs* and *NLRs* in triggering the inflammatory signaling and inflammasome activation, respectively, during NP, and this resulted in placental inflammation and immune cell chemotaxis. The increased leukocytic infiltration was associated with the upregulation of *MMPs* and apoptosis-related genes, believed to be implicated in placental separation during NP. NP was associated with aberrant expression of several placenta-regulatory genes and dysregulation of placental angiogenesis, and nutrient transportation, as well as placental hypoxia, which could explain placental insufficiency during NP. The significant findings of the current dataset are summarized in Figure 11. Beyond the NP-associated events described in the current study, this study provides a comprehensive database of the key upstream regulators, TFs, hub genes, receptor-ligand interactions, gene co-expression networks associated with equine nocardioform placentitis. Strategies to block the key upstream regulators (e.g., TLRs and NLRs) and associated pathways hold potential for therapies to overcome NP. Together, the currently revealed placentitis transcriptome signature improves our understanding of the disease. Also, it provides an all-essential basis for the construction of new hypotheses to be tested in the future. Finally, much more valuable information remains to be mined from the current transcriptome dataset.

## Supplementary Information

**Additional file 1. Summary of the bioinformatics and functional genomics pipeline used in the current study.**

**Additional file 2. Clinical and pathological findings in for all mares included in the current study.**

**Additional file 3. Read counts and mapping quality of Illumina RNA-sequencing dataset from equine chorioallantois (CA) retrieved from CRL, NPL, and NPN.** Key; CRL; normal postpartum (control), NPL; nocardioform placentitis lesion, and NPN; nocardioform placentitis normal region.

**Additional file 4. Differentially expressed genes (DEGs) in equine chorioallantois during nocardioform placentitis (NPL vs. CRL and NPL vs. NPN sets).** Key; CRL; normal postpartum (control), NPL; nocardioform placentitis lesion, and NPN; nocardioform placentitis normal region.

**Additional file 5. Potential upstream regulators in equine chorioallantois (CA) during nocardioform placentitis (NP) as identified by upstream regulator analysis performed on Ingenuity Pathway Analysis (IPA) using DEG**_**S**_** in NPL vs. CRL set (A) and DEG**_**S**_** in NPL vs. NPN set (B).** Key; CRL; normal postpartum (control) and NPL; nocardioform placentitis lesion.

**Additional file 6. Weighted gene expression co-expression network analysis (WGCNA) of DEGs in equine chorioallantois (CA) during nocardioform placentitis dataset.** A) DEGs and corresponding module (cluster) as generated by WGCNA. B) Intramodular hub genes in the turquoise module.

**Additional file 7. List of predicted ligand and receptor pairs and their expression patterns in equine chorioallantois (CA) during nocardioform placentitis dataset.**

## Data Availability

The current RNA-seq data was deposited in the Gene Expression Omnibus (GEO; GSE154637) repository.
