# Supplementary figures and images for: Transcriptomic analysis of equine chorioallantois reveals immune networks and molecular mechanisms involved in nocardioform placentitis
Source: Vet Res. 2021 Jul 8;52:103. doi: 10.1186/s13567-021-00972-4 (PMC8268225; doi:10.1186/s13567-021-00972-4)

## Slide 1
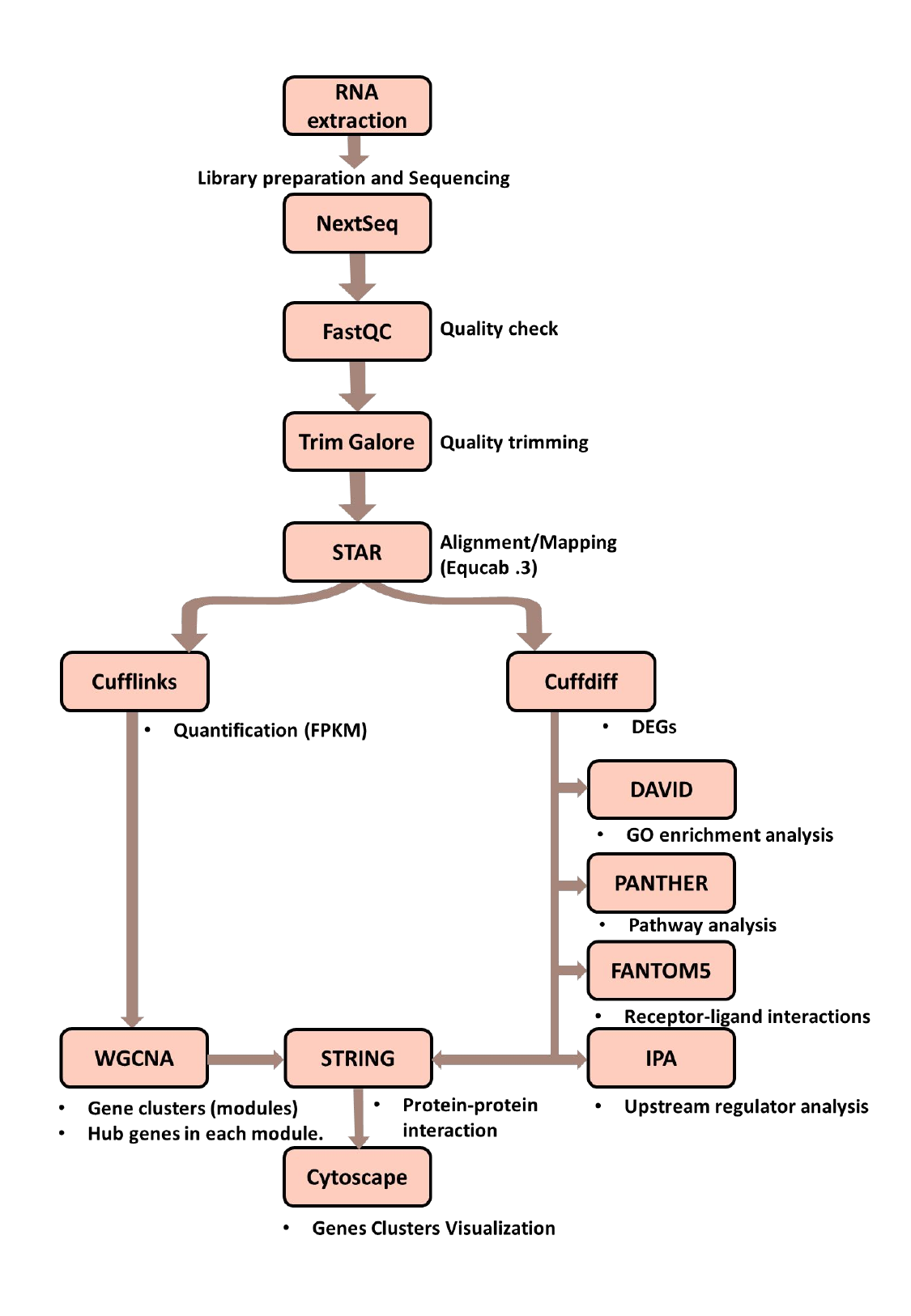

Supplement: Supplementary file 1 — Additional file 1. Summary of the bioinformatics and functional genomics pipeline used in the current study. [file 13567_2021_972_MOESM1_ESM.pptx]
